# Reclassification of Whole Exome Sequencing-derived Genetic Variants in Pendred Syndrome with ACMG/AMP Standards

**DOI:** 10.1055/s-0041-1725072

**Published:** 2021-03-10

**Authors:** Kok-Siong Poon, Karen Mei-Ling Tan

**Affiliations:** 1 Department of Laboratory Medicine, National University Hospital Singapore, Singapore, Singapore

Whole exome sequencing (WES) opens up an unbiased testing approach for genetic diagnosis of rare disorders compared with single-gene and multigene panel testing. Being a diagnostic tool for a wide range of genetic diseases, WES identifies causal variants in close to one-third of the referred cases, and variants of unknown significance (VUS) potentially responsible for the clinical presentations in another one quarter of index patients. 1 Systematic guidelines from Human Genome Variation Society (HGVS), and American College of Medical Genetics and Genomics (ACMG) and Association for Molecular Pathology (AMP) are currently available for standardizing the nomenclature and reporting the interpretation of genetic variants, respectively. 2 3 Despite this, variant curation still remains a challenge in the face of advances in sequencing technologies, which may reveal large numbers of novel variants possibly associated with the clinical phenotype in the diagnostic setting. Depending on the disease context, reanalysis of WES-derived genetic variants can sometimes improve diagnostic yields 4 or result in downgrading of the pathogenicity status of some previously reported variants. 5

Pendred syndrome (OMIM 274600) is an autosomal recessive disorder characterized by sensorineural hearing loss, goiter, and inner ear malformations. It is most commonly caused by biallelic loss-of-function variants in the *SLC26A4* gene. 6 Digenic inheritance of monoallelic loss-of-function variants in the *SLC26A4* gene and another gene, for example, either *KCNJ10* , *FOXI1* or *GJB2* have also been described. 7 8 9 However, subsequent studies refuted the causal associations between *KCNJ10* or *FOXI1* mutations and *SLC26A4* mutations in this syndrome. 10 11

Here, we demonstrated the reclassification of causative variants previously reported in a study 11 in which WES was performed on a family with two daughters clinically diagnosed with Pendred syndrome and their unaffected parents. 12 On the basis of possible autosomal recessive, digenic or oligogenic modes of inheritance, pathogenic variants were sought in the two sisters. Thyroid dysfunction in the two siblings was attributed to compound heterozygous variants (p.Lys350* and p.Arg1110Gln) in the *DUOX2* gene. Interestingly, Chow et al proposed that the combination of heterozygous variants in three genes ( *SLC26A4* p.Ser448Leu, *GJB2* p.Thr123Asn, and *SCARB2* p.Thr305Met), which were found in both affected siblings, led to bilateral hearing loss.

Digenic inheritance usually involves pathogenic variants in genes encoding interacting proteins, and results in a “double hit” affecting the function or structure of the protein complex. 13 Notably, the three genes reported by Chow et al, namely, *SLC26A4* , *GJB2* and *SCARB2* , have no known protein interactions between their gene products. In Chow et al, the evidence of pathogenicity for the candidate variants in these genes were derived solely from *in silico* predictions, without evidence from functional analyses or gene-disease associations other than from the single family. This prompted us to further curate these variants according to the ACMG/AMP standards 3 using VarSome. 14 We also performed data mining in ClinVar, a database commonly referenced for germline variants. 15 The pathogenicity of the variants identified in the two siblings with Pendred syndrome in Chow et al are shown in Table 1 . After re-evaluation, the *SLC26A4* NM_000441.2:c.1343C > T variant was classified as “pathogenic” and “likely pathogenic” by ACMG/AMP and ClinVar, respectively. Strikingly, the *GJB2* NM_004004.6:c.368C > A and *SCARB2* NM_005506.4:c.914C > T variants were classified as either benign or VUS. The *GJB2* p.Thr123Asn variant has been detected at higher frequencies in controls compared with patients 16 and identified in *trans* with pathogenic variants in the *GJB2* gene in normal carriers. 17 Taking these reevaluations into consideration, the *GJB2* and *SCARB2* variants are now “downgraded” in their pathogenicity status and unlikely to be associated with deafness in concert with the *SLC26A4* variant.

**Table 1:** Classification of variants according to the ACMG/AMP standards and ClinVar

Gene	Variant with HGVS nomenclature		ACMG/AMP classification by VarSome version 8.4.7			ClinVar	
	mRNA level	Protein level	Interpretation	Automated criteria	Short link	Interpretation	Variation ID
*SLC26A4*	NM_000441.2:c.1343C > T	p.Ser448Leu	Pathogenic	PM1, PM2, PM5, PP2, PP3, PP5.	http://varso.me/H0yK	Likely pathogenic	549979
*GJB2*	NM_004004.6:c.368C > A	p.Thr123Asn	Benign	PM1, PM2, PP2, BP4, BP6	http://varso.me/Fe5Y	Benign/Likely benign	44743
*SCARB2*	NM_005506.4:c.914C > T	p.Thr305Met	Likely benign	PM1, PM2, BP1, BP4	http://varso.me/OOe7	Uncertain significance	588761
*DUOX2*	NM_014080.4:c.1588A > T	p.Lys530*	Pathogenic	PVS1, PM2, PP3, PP5	http://varso.me/EuGP	Pathogenic	287079
*DUOX2*	NM_014080.4:c.3329G > A	p.Arg1110Gln	Pathogenic	PM1, PM2, PP2, PP3, PP5.	http://varso.me/Eozb	Pathogenic	420157

On the other hand, the two *DUOX2* variants, namely, NM_014080.4:c.1588A > T and c.3329G > A were classified as “pathogenic” in the context of goiter by ACMG/AMP standards and in ClinVar. These classifications are consistent with Chow et al's interpretations. The two pathogenic variants in the *DUOX2* gene identified (p.Lys350* and p.Arg1110Gln) have been found in patients with congenital hypothyroidism. 18 19 20

WES investigates all coding exons in a relatively time-efficient and cost-effective manner compared with the traditional single-gene or multigene panel testing. It is not unusual that many rare variants can be identified in multiple genes by WES. It remains a big challenge to firmly assign causal variants and genes when there is only one individual or a single family, especially in complex diseases with possibly digenic or polygenic inheritance. There are many essential points to consider when evaluating the pathogenicity of variants 3 and also when conducting reevaluation and reanalysis of genomic test results. 21 Having timely and accurately updated public databases of variants is essential to the research and clinical diagnostic communities for variant curation and clinical diagnosis.

## References

[1] Trujillano D Bertoli-Avella A M Kumar Kandaswamy K Clinical exome sequencing: results from 2819 samples reflecting 1000 families Eur J Hum Genet 2017 25 02 176 182 27848944 10.1038/ejhg.2016.146 PMC5255946

[2] den Dunnen J T Dalgleish R Maglott D R HGVS recommendations for the description of sequence variants: 2016 update Hum Mutat 2016 37 06 564 569 26931183 10.1002/humu.22981

[3] ACMG Laboratory Quality Assurance Committee Richards S Aziz N Bale S Standards and guidelines for the interpretation of sequence variants: a joint consensus recommendation of the American College of Medical Genetics and Genomics and the Association for Molecular Pathology Genet Med 2015 17 05 405 424 25741868 10.1038/gim.2015.30 PMC4544753

[4] Salfati E L Spencer E G Topol S E Re-analysis of whole-exome sequencing data uncovers novel diagnostic variants and improves molecular diagnostic yields for sudden death and idiopathic diseases Genome Med 2019 11 01 83 31847883 10.1186/s13073-019-0702-2 PMC6916453

[5] Campuzano O Sarquella-Brugada G Fernandez-Falgueras A Reanalysis and reclassification of rare genetic variants associated with inherited arrhythmogenic syndromes EBioMedicine 2020 54 102732 32268277 10.1016/j.ebiom.2020.102732 PMC7136601

[6] Bizhanova A Kopp P Genetics and phenomics of Pendred syndrome Mol Cell Endocrinol 2010 322(1-2):83 90 20298745 10.1016/j.mce.2010.03.006

[7] Yang T Vidarsson H Rodrigo-Blomqvist S Rosengren S S Enerback S Smith R J Transcriptional control of SLC26A4 is involved in Pendred syndrome and nonsyndromic enlargement of vestibular aqueduct (DFNB4) Am J Hum Genet 2007 80 06 1055 1063 17503324 10.1086/518314 PMC1867094

[8] Yang T Gurrola J G II Wu H Mutations of KCNJ10 together with mutations of SLC26A4 cause digenic nonsyndromic hearing loss associated with enlarged vestibular aqueduct syndrome Am J Hum Genet 2009 84 05 651 657 19426954 10.1016/j.ajhg.2009.04.014 PMC2681005

[9] Ben Said M Dhouib H BenZina Z Segregation of a new mutation in SLC26A4 and p.E47X mutation in GJB2 within a consanguineous Tunisian family affected with Pendred syndrome Int J Pediatr Otorhinolaryngol 2012 76 06 832 836 22429511 10.1016/j.ijporl.2012.02.053

[10] Landa P Differ A M Rajput K Jenkins L Bitner-Glindzicz M Lack of significant association between mutations of KCNJ10 or FOXI1 and SLC26A4 mutations in Pendred syndrome/enlarged vestibular aqueducts BMC Med Genet 2013 14 85 23965030 10.1186/1471-2350-14-85 PMC3765178

[11] Zhao J Yuan Y Huang S KCNJ10 may not be a contributor to nonsyndromic enlargement of vestibular aqueduct (NSEVA) in Chinese subjects PLoS One 2014 9 11 e108134 25372295 10.1371/journal.pone.0108134 PMC4220913

[12] Chow Y P Abdul Murad N A Mohd Rani Z Exome sequencing identifies SLC26A4, GJB2, SCARB2 and DUOX2 mutations in 2 siblings with Pendred syndrome in a Malaysian family Orphanet J Rare Dis 2017 12 01 40 28222800 10.1186/s13023-017-0575-7 PMC5320863

[13] Schäffer A A Digenic inheritance in medical genetics J Med Genet 2013 50 10 641 652 23785127 10.1136/jmedgenet-2013-101713 PMC3778050

[14] Kopanos C Tsiolkas V Kouris A VarSome: the human genomic variant search engine Bioinformatics 2019 35 11 1978 1980 30376034 10.1093/bioinformatics/bty897 PMC6546127

[15] Landrum M J Lee J M Benson M ClinVar: improving access to variant interpretations and supporting evidence Nucleic Acids Res 2018 46(D1):D1062 D1067 29165669 10.1093/nar/gkx1153 PMC5753237

[16] Dai P Yu F Han B GJB2 mutation spectrum in 2,063 Chinese patients with nonsyndromic hearing impairment J Transl Med 2009 7 26 19366456 10.1186/1479-5876-7-26 PMC2679712

[17] He L Pang X Chen P Wang X Yang T Wu H Carrier re-sequencing reveals rare but benign variants in recessive deafness genes Sci Rep 2017 7 01 11355 28900111 10.1038/s41598-017-10099-2 PMC5595904

[18] Fu C Zhang S Su J Mutation screening of DUOX2 in Chinese patients with congenital hypothyroidism J Endocrinol Invest 2015 38 11 1219 1224 26349762 10.1007/s40618-015-0382-8

[19] Maruo Y Takahashi H Soeda I Transient congenital hypothyroidism caused by biallelic mutations of the dual oxidase 2 gene in Japanese patients detected by a neonatal screening program J Clin Endocrinol Metab 2008 93 11 4261 4267 18765513 10.1210/jc.2008-0856

[20] Fu C Luo S Zhang S Next-generation sequencing analysis of DUOX2 in 192 Chinese subclinical congenital hypothyroidism (SCH) and CH patients Clin Chim Acta 2016 458 30 34 27108200 10.1016/j.cca.2016.04.019

[21] ACMG Laboratory Quality Assurance Committee Deignan J L Chung W K Kearney H M Monaghan K G Rehder C W Chao E C Points to consider in the reevaluation and reanalysis of genomic test results: a statement of the American College of Medical Genetics and Genomics (ACMG) Genet Med 2019 21 06 1267 1270 31015575 10.1038/s41436-019-0478-1 PMC6559819

